# The genome sequence of the Thicket Knot-horn, *Acrobasis suavella *(Zincken, 1818)

**DOI:** 10.12688/wellcomeopenres.19506.1

**Published:** 2023-06-19

**Authors:** Douglas Boyes, James Hammond, University of Oxford and Wytham Woods Genome Acquisition Lab, Darwin Tree of Life Barcoding collective, Wellcome Sanger Institute Tree of Life programme, Wellcome Sanger Institute Scientific Operations: DNA Pipelines collective, Tree of Life Core Informatics collective, Darwin Tree of Life Consortium

**Affiliations:** 1 UK Centre for Ecology & Hydrology, Wallingford, England, UK; 2 University of Oxford, Oxford, England, UK

**Keywords:** Acrobasis suavella, Thicket Knot-horn, genome sequence, chromosomal, Lepidoptera

## Abstract

We present a genome assembly from an individual male *Acrobasis suavella* (the Thicket Knot-horn; Arthropoda; Insecta; Lepidoptera; Pyralidae). The genome sequence is 647.3 megabases in span. Most of the assembly is scaffolded into 30 chromosomal pseudomolecules, including the Z sex chromosome. The mitochondrial genome has also been assembled and is 15.31 kilobases in length. Gene annotation of this assembly on Ensembl identified 19,101 protein coding genes.

## Species taxonomy

Eukaryota; Metazoa; Eumetazoa; Bilateria; Protostomia; Ecdysozoa; Panarthropoda; Arthropoda; Mandibulata; Pancrustacea; Hexapoda; Insecta; Dicondylia; Pterygota; Neoptera; Endopterygota; Amphiesmenoptera; Lepidoptera; Glossata; Neolepidoptera; Heteroneura; Ditrysia; Obtectomera; Pyraloidea; Pyralidae; Phycitinae; *Acrobasis*; *Acrobasis suavella* (Zincken, 1818) (NCBI:txid1857951).

## Background

*Acrobasis suavella* (Zincken, 1818) is a moth of the Pyralidae family. The adult moths of this species are marked with a mixture of ruddy purple and grey on the forewings, and in some specimens the intensity of these markings can create a handsome burgundy and silver appearance to the moth. The adults of this species are on the wing in Britain and Ireland between June and August, flying at night. The adult moth is seldom seen by day but comes readily to light ( Goater *et al.*, 1986; Parsons & Davis, 2018)

The most frequently recorded larval foodplant for the species in Britain and Ireland is *Prunus spinosa*, but larvae have been found on *Cotoneaster*, *Crataegus*, and *Sorbus* ( Parsons & Davis, 2018). The species reportedly prefers stunted and isolated *P. spinosa* plants, and open habitats such as downland where such plants occur ( Goater *et al.*, 1986; Parsons & Davis, 2018). The larva feeds from September to June within a thick silken tube coated with leaf fragments and larval frass, and pupation occurs within, or adjacent to, the larval gallery ( Parsons & Davis, 2018).

In Britain, the moth is most widespread across southern England and Wales, but there is also a record from Shetland ( Langmaid & Young, 2004), possibly indicating vagrancy. Globally the species is found across Europe east to the Caucasus ( Streltzov *et al.*, 2022), and appears to have become established in North America, around Vancouver, British Columbia, since at least the early 20th century, feeding on Cotoneaster ( Heinrich, 1939; Neunzig, 1990). It is therefore possible the species may expand its range in the future via the ornamental plants trade.

The genome of *Acrobasis suavella* was sequenced as part of the Darwin Tree of Life Project, a collaborative effort to sequence all named eukaryotic species in the Atlantic Archipelago of Britain and Ireland. Here we present a chromosomally complete genome sequence for *Acrobasis suavella*, based on one male specimen from Wytham Woods, Oxfordshire, UK.

## Genome sequence report

**Figure 1.**
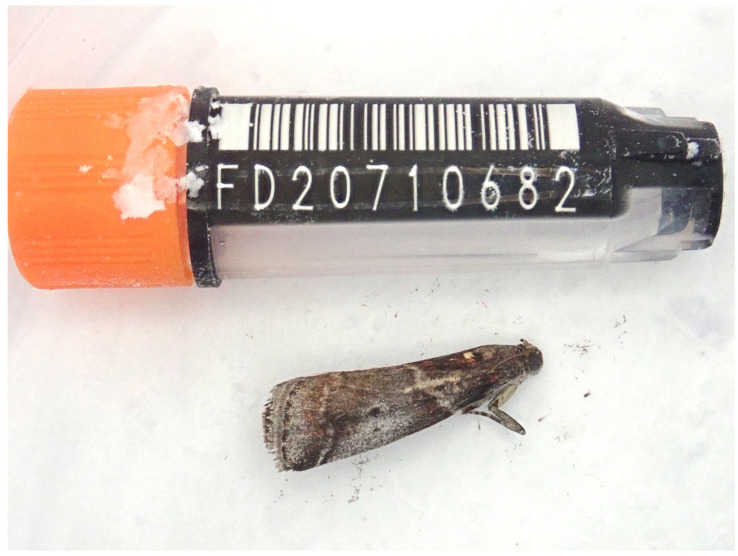
Photograph of the *Acrobasis suavella* (ilAcrSuav1) specimen used for genome sequencing.

The genome was sequenced from one male *Acrobasis suavella* ( Figure 1) collected from Wytham Woods, Oxfordshire, UK (51.77, –1.34). A total of 26-fold coverage in Pacific Biosciences single-molecule HiFi long reads was generated. Primary assembly contigs were scaffolded with chromosome conformation Hi-C data. Manual assembly curation corrected 7 missing joins or mis-joins and removed one haplotypic duplication, reducing the assembly length by 0.13%% and the scaffold number by 11.43%.

**Figure 2.**
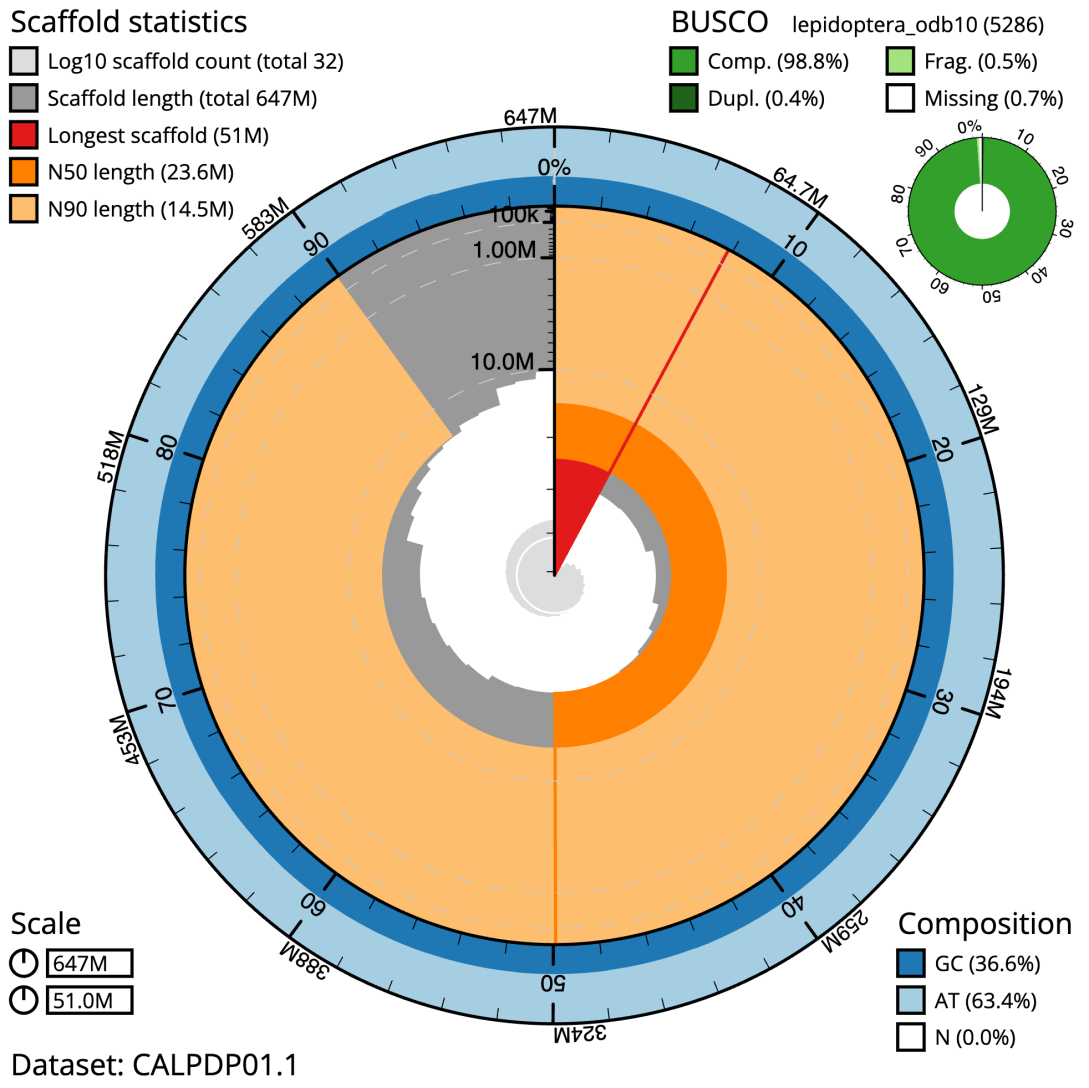
Genome assembly of *Acrobasis suavella*, ilAcrSuav1.1: metrics. The BlobToolKit Snailplot shows N50 metrics and BUSCO gene completeness. The main plot is divided into 1,000 size-ordered bins around the circumference with each bin representing 0.1% of the 647,282,432 bp assembly. The distribution of scaffold lengths is shown in dark grey with the plot radius scaled to the longest scaffold present in the assembly (51,000,710 bp, shown in red). Orange and pale-orange arcs show the N50 and N90 scaffold lengths (23,584,496 and 14,517,006 bp), respectively. The pale grey spiral shows the cumulative scaffold count on a log scale with white scale lines showing successive orders of magnitude. The blue and pale-blue area around the outside of the plot shows the distribution of GC, AT and N percentages in the same bins as the inner plot. A summary of complete, fragmented, duplicated and missing BUSCO genes in the lepidoptera_odb10 set is shown in the top right. An interactive version of this figure is available at https://blobtoolkit.genomehubs.org/view/ilAcrSuav1.1/dataset/CALPDP01.1/snail.

The final assembly has a total length of 647.3 Mb in 31 sequence scaffolds with a scaffold N50 of 23.6 Mb ( Table 1). Most (99.99%) of the assembly sequence was assigned to 30 chromosomal-level scaffolds, representing 29 autosomes and the Z sex chromosome. Chromosome-scale scaffolds confirmed by the Hi-C data are named in order of size ( Figure 2– Figure 5; Table 2). While not fully phased, the assembly deposited is of one haplotype. Contigs corresponding to the second haplotype have also been deposited. The mitochondrial genome was also assembled and can be found as a contig within the multifasta file of the genome submission.

**Table 1.** Genome data for *Acrobasis suavella*, ilAcrSuav1.1.

Project accession data		
Assembly identifier	ilAcrSuav1.1	
Species	*Acrobasis suavella*	
Specimen	ilAcrSuav1	
NCBI taxonomy ID	1857951	
BioProject	PRJEB52024	
BioSample ID	SAMEA10979088	
Isolate information	ilAcrSuav1, male: whole organism (DNA sequencing and HiC scaffolding) ilAcrSuav3: whole organism (RNA sequencing)	
Assembly metrics *		*Benchmark*
Consensus quality (QV)	63.6	*≥ 50*
*k*-mer completeness	100%	*≥ 95%*
BUSCO **	C:98.8%[S:98.4%,D:0.4%], F:0.5%,M:0.7%,n:5,286	*C ≥ 95%*
Percentage of assembly mapped to chromosomes	99.99%	*≥ 95%*
Sex chromosomes	Z chromosome	*localised homologous pairs*
Organelles	Mitochondrial genome assembled	*complete single alleles*
Raw data accessions		
PacificBiosciences SEQUEL II	ERR9745002	
Hi-C Illumina	ERR9503461	
PolyA RNA-Seq Illumina	ERR10123692	
Genome assembly		
Assembly accession	GCA_943193695.1	
*Accession of alternate haplotype*	GCA_943193685.1	
Span (Mb)	647.3	
Number of contigs	54	
Contig N50 length (Mb)	22.0	
Number of scaffolds	31	
Scaffold N50 length (Mb)	23.6	
Longest scaffold (Mb)	51.0	
Genome annotation		
Number of protein-coding genes	19,101	
Number of gene transcripts	19,275	

* Assembly metric benchmarks are adapted from column VGP-2020 of “Table 1: Proposed standards and metrics for defining genome assembly quality” from ( Rhie *et al.*, 2021). ** BUSCO scores based on the lepidoptera_odb10 BUSCO set using v5.3.2. C = complete [S = single copy, D = duplicated], F = fragmented, M = missing, n = number of orthologues in comparison. A full set of BUSCO scores is available at https://blobtoolkit.genomehubs.org/view/ilAcrSuav1.1/dataset/CALPDP01.1/busco.

**Figure 3.**
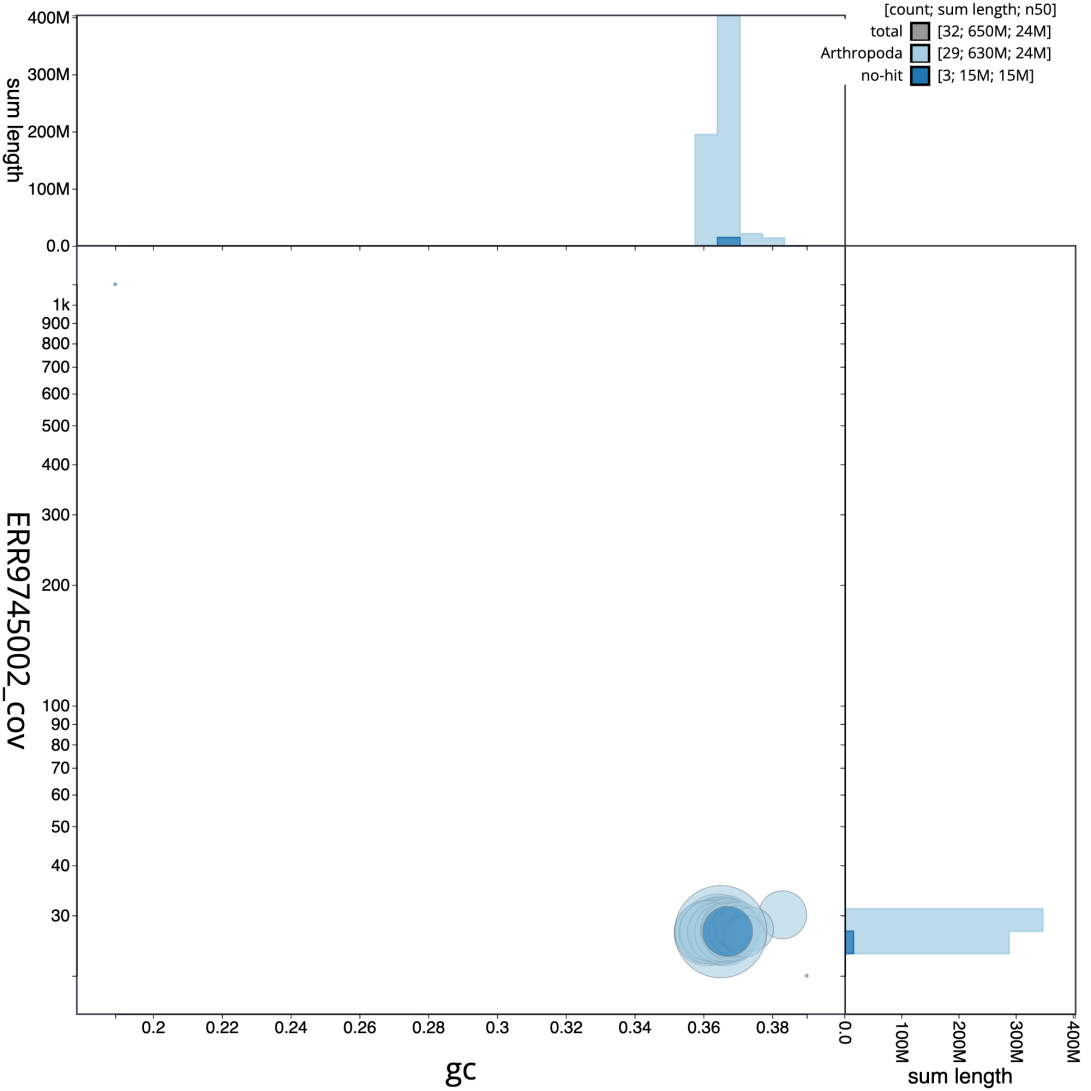
Genome assembly of *Acrobasis suavella*, ilAcrSuav1.1: BlobToolKit GC-coverage plot. Scaffolds are coloured by phylum. Circles are sized in proportion to scaffold length. Histograms show the distribution of scaffold length sum along each axis. An interactive version of this figure is available at https://blobtoolkit.genomehubs.org/view/ilAcrSuav1.1/dataset/CALPDP01.1/blob.

**Figure 4.**
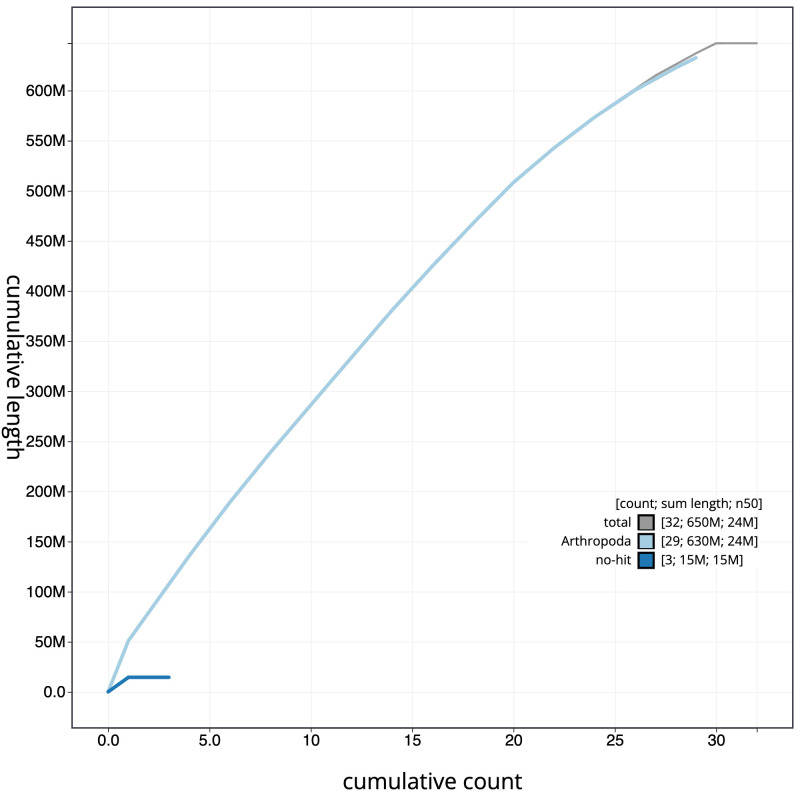
Genome assembly of *Acrobasis suavella*, ilAcrSuav1.1: BlobToolKit cumulative sequence plot. The grey line shows cumulative length for all scaffolds. Coloured lines show cumulative lengths of scaffolds assigned to each phylum using the buscogenes taxrule. An interactive version of this figure is available at https://blobtoolkit.genomehubs.org/view/ilAcrSuav1.1/dataset/CALPDP01.1/cumulative.

**Figure 5.**
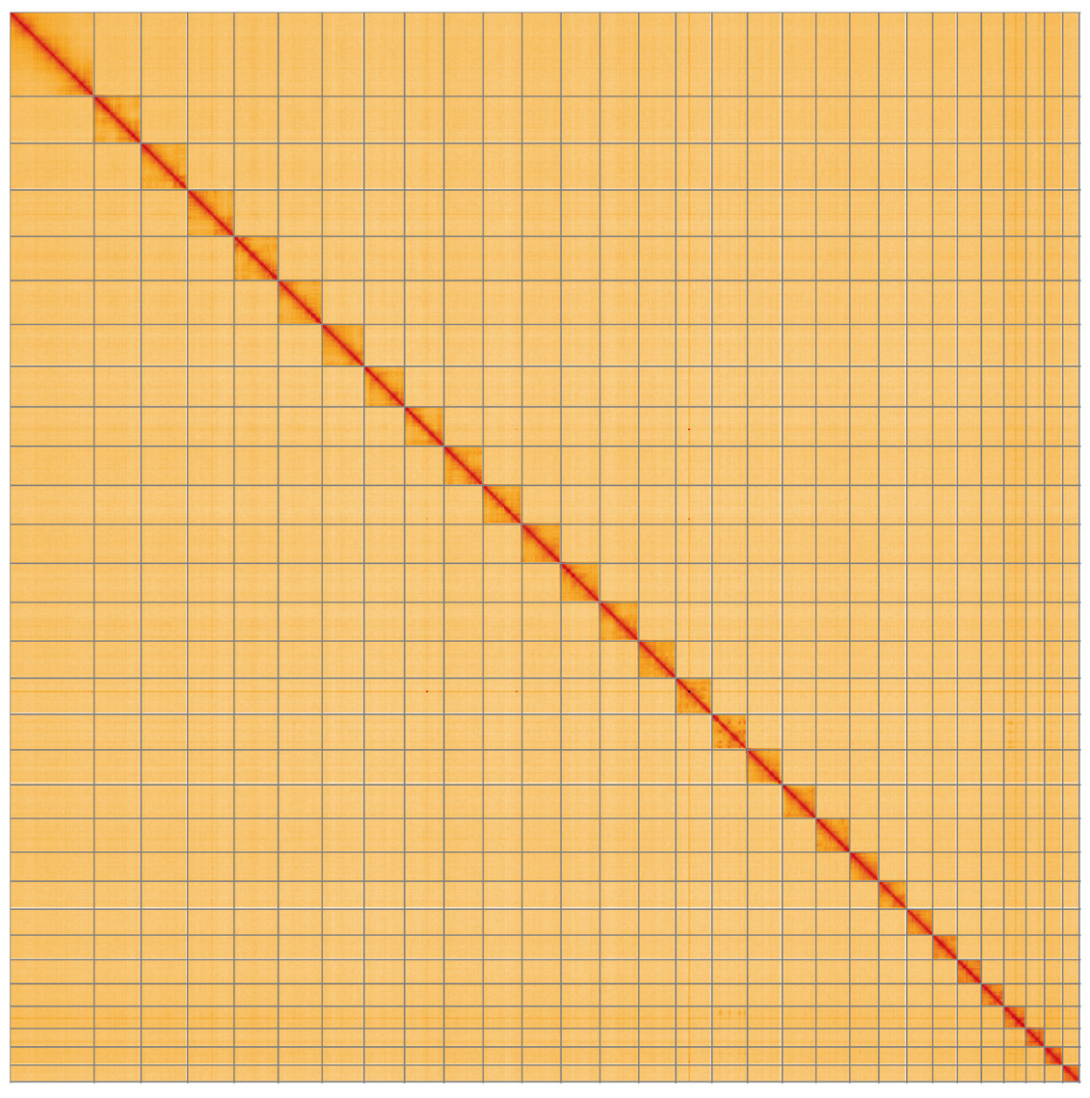
Genome assembly of *Acrobasis suavella*, ilAcrSuav1.1: Hi-C contact map of the ilAcrSuav1.1 assembly, visualised using HiGlass. Chromosomes are shown in order of size from left to right and top to bottom. An interactive version of this figure may be viewed at https://genome-note-higlass.tol.sanger.ac.uk/l/?d=W69aQoBuSxGnPnFq02aE5Q.

**Table 2.** Chromosomal pseudomolecules in the genome assembly of *Acrobasis suavella*, ilAcrSuav1.

INSDC accession	Chromosome	Length (Mb)	GC%
OW971929.1	1	28.4	36.5
OW971930.1	2	28.2	36.5
OW971931.1	3	28.13	36.5
OW971932.1	4	26.65	36.5
OW971933.1	5	26.64	36.5
OW971934.1	6	25.35	36.0
OW971935.1	7	24.43	36.5
OW971936.1	8	23.91	36.5
OW971937.1	9	23.71	36.5
OW971938.1	10	23.6	36.0
OW971939.1	11	23.58	36.5
OW971940.1	12	23.58	36.0
OW971941.1	13	23.45	36.5
OW971942.1	14	22.38	36.5
OW971943.1	15	21.95	36.0
OW971944.1	16	21.48	36.5
OW971945.1	17	21.05	36.5
OW971946.1	18	20.46	36.5
OW971947.1	19	20.43	37.0
OW971948.1	20	17.55	36.5
OW971949.1	21	16.84	36.5
OW971950.1	22	15.74	36.5
OW971951.1	23	14.92	37.0
OW971952.1	24	14.52	36.5
OW971953.1	25	13.66	37.0
OW971954.1	26	13.43	38.5
OW971955.1	27	11.18	37.0
OW971956.1	28	10.95	37.5
OW971957.1	29	10.06	37.0
OW971928.1	Z	51.0	36.5
OW971958.1	MT	0.02	19.5

The estimated Quality Value (QV) of the final assembly is 63.6 with *k*-mer completeness of 100%, and the assembly has a BUSCO v5.3.2 completeness of 98.8% (single = 98.4%, duplicated = 0.4%), using the lepidoptera_odb10reference set ( *n* = 5,286).

Metadata for specimens, spectral estimates, sequencing runs, contaminants and pre-curation assembly statistics can be found at https://links.tol.sanger.ac.uk/species/1857951.

## Genome annotation report

The *Acrobasis suavella* genome assembly (GCA_943193695.1) was annotated using the Ensembl rapid annotation pipeline ( Table 1; https://rapid.ensembl.org/Acrobasis_suavella_GCA_943193695.1/Info/Index). The resulting annotation includes 19,275 transcribed mRNAs from 19,101 protein-coding genes.

## Methods

### Sample acquisition and nucleic acid extraction

Two *Acrobasis suavella* specimens (ilAcrSuav1 and ilAcrSuav3) were collected from Wytham Woods, Oxfordshire (biological vice-county Berkshire), UK (latitude 51.77, longitude –1.34) on 2021-07-24. The specimens were taken from a grassland habitat using a light trap. The specimens were collected and identified by Douglas Boyes (University of Oxford) and were snap-frozen on dry ice.

DNA was extracted at the Tree of Life laboratory, Wellcome Sanger Institute (WSI). The ilAcrSuav1 sample was weighed and dissected on dry ice with tissue set aside for Hi-C sequencing. Whole organism tissue was disrupted using a Nippi Powermasher fitted with a BioMasher pestle. High molecular weight (HMW) DNA was extracted using the Qiagen MagAttract HMW DNA extraction kit. HMW DNA was sheared into an average fragment size of 12–20 kb in a Megaruptor 3 system with speed setting 30. Sheared DNA was purified by solid-phase reversible immobilisation using AMPure PB beads with a 1.8X ratio of beads to sample to remove the shorter fragments and concentrate the DNA sample. The concentration of the sheared and purified DNA was assessed using a Nanodrop spectrophotometer and Qubit Fluorometer and Qubit dsDNA High Sensitivity Assay kit. Fragment size distribution was evaluated by running the sample on the FemtoPulse system.

RNA was extracted from whole organism tissue of ilAcrSuav3 in the Tree of Life Laboratory at the WSI using TRIzol, according to the manufacturer’s instructions. RNA was then eluted in 50 μl RNAse-free water and its concentration assessed using a Nanodrop spectrophotometer and Qubit Fluorometer using the Qubit RNA Broad-Range (BR) Assay kit. Analysis of the integrity of the RNA was done using Agilent RNA 6000 Pico Kit and Eukaryotic Total RNA assay.

### Sequencing

Pacific Biosciences HiFi circular consensus DNA sequencing libraries were constructed according to the manufacturers’ instructions. Poly(A) RNA-Seq libraries were constructed using the NEB Ultra II RNA Library Prep kit. DNA and RNA sequencing was performed by the Scientific Operations core at the WSI on Pacific Biosciences SEQUEL II (HiFi) and Illumina NovaSeq 6000 (RNA-Seq) instruments. Hi-C data were also generated from tissue of ilAcrSuav1 that had been set aside, using the Arima2 kit and sequenced on the Illumina NovaSeq 6000 instrument.

### Genome assembly, curation and evaluation

Assembly was carried out with Hifiasm ( Cheng *et al.*, 2021) and haplotypic duplication was identified and removed with purge_dups ( Guan *et al.*, 2020). The assembly was scaffolded with Hi-C data ( Rao *et al.*, 2014) using YaHS ( Zhou *et al.,* 2023). The assembly was checked for contamination as described previously ( Howe *et al.*, 2021). Manual curation was performed using HiGlass ( Kerpedjiev *et al.*, 2018) and Pretext ( Harry, 2022). The mitochondrial genome was assembled using MitoHiFi ( Uliano-Silva *et al.*, 2022), which runs MitoFinder ( Allio *et al.*, 2020) or MITOS ( Bernt *et al.*, 2013) and uses these annotations to select the final mitochondrial contig and to ensure the general quality of the sequence.

A Hi-C map for the final assembly was produced using bwa-mem2 ( Vasimuddin *et al.*, 2019) in the Cooler file format ( Abdennur & Mirny, 2020). To assess the assembly metrics, the *k*-mer completeness and QV consensus quality values were calculated in Merqury ( Rhie *et al.*, 2020). This work was done using Nextflow ( Di Tommaso *et al.*, 2017) DSL2 pipelines “sanger-tol/readmapping” ( Surana *et al.,* 2023a) and “sanger-tol/genomenote” ( Surana *et al.,* 2023b). The genome was analysed within the BlobToolKit environment ( Challis *et al.*, 2020) and BUSCO scores ( Manni *et al.*, 2021; Simão *et al.*, 2015) were calculated.

Table 3 contains a list of relevant software tool versions and sources.

**Table 3.** Software tools: versions and sources.

Software tool	Version	Source
BlobToolKit	4.1.3	https://github.com/blobtoolkit/blobtoolkit
BUSCO	5.3.2	https://gitlab.com/ezlab/busco
Hifiasm	0.16.1-r375	https://github.com/chhylp123/hifiasm
HiGlass	1.11.6	https://github.com/higlass/higlass
Merqury	MerquryFK	https://github.com/thegenemyers/MERQURY.FK
MitoHiFi	2	https://github.com/marcelauliano/MitoHiFi
PretextView	0.2	https://github.com/wtsi-hpag/PretextView
purge_dups	1.2.3	https://github.com/dfguan/purge_dups
sanger-tol/genomenote	v1.0	https://github.com/sanger-tol/genomenote
sanger-tol/readmapping	1.1.0	https://github.com/sanger-tol/readmapping/tree/1.1.0
YaHS	yahs-1.1.91eebc2	https://github.com/c-zhou/yahs

### Genome annotation

The BRAKER2 pipeline ( Brůna *et al.*, 2021) was used in the default protein mode to generate annotation for the *Acrobasis suavella* assembly (GCA_943193695.1) in Ensembl Rapid Release.

### Wellcome Sanger Institute – Legal and Governance

The materials that have contributed to this genome note have been supplied by a Darwin Tree of Life Partner.

The submission of materials by a Darwin Tree of Life Partner is subject to the **‘Darwin Tree of Life Project Sampling Code of Practice’**, which can be found in full on the Darwin Tree of Life website here. By agreeing with and signing up to the Sampling Code of Practice, the Darwin Tree of Life Partner agrees they will meet the legal and ethical requirements and standards set out within this document in respect of all samples acquired for, and supplied to, the Darwin Tree of Life Project.

Further, the Wellcome Sanger Institute employs a process whereby due diligence is carried out proportionate to the nature of the materials themselves, and the circumstances under which they have been/are to be collected and provided for use. The purpose of this is to address and mitigate any potential legal and/or ethical implications of receipt and use of the materials as part of the research project, and to ensure that in doing so we align with best practice wherever possible.

The overarching areas of consideration are:

- Ethical review of provenance and sourcing of the material
- Legality of collection, transfer and use (national and international)

Each transfer of samples is further undertaken according to a Research Collaboration Agreement or Material Transfer Agreement entered into by the Darwin Tree of Life Partner, Genome Research Limited (operating as the Wellcome Sanger Institute), and in some circumstances other Darwin Tree of Life collaborators.

## Data Availability

European Nucleotide Archive: *Acrobasis suavella* (thicket knot-horn). Accession number PRJEB52024; https://identifiers.org/ena.embl/PRJEB52024. ( Wellcome Sanger Institute, 2022) The genome sequence is released openly for reuse. The *Acrobasis suavella* genome sequencing initiative is part of the Darwin Tree of Life (DToL) project. All raw sequence data and the assembly have been deposited in INSDC databases. Raw data and assembly accession identifiers are reported in Table 1.

## Author information

Members of the University of Oxford and Wytham Woods Genome Acquisition Lab are listed here: https://doi.org/10.5281/zenodo.4789928.

Members of the Darwin Tree of Life Barcoding collective are listed here: https://doi.org/10.5281/zenodo.4893703.

Members of the Wellcome Sanger Institute Tree of Life programme are listed here: https://doi.org/10.5281/zenodo.4783585.

Members of Wellcome Sanger Institute Scientific Operations: DNA Pipelines collective are listed here: https://doi.org/10.5281/zenodo.4790455.

Members of the Tree of Life Core Informatics collective are listed here: https://doi.org/10.5281/zenodo.5013541.

Members of the Darwin Tree of Life Consortium are listed here: https://doi.org/10.5281/zenodo.4783558.

## References

[ref-1] Abdennur N Mirny LA : Cooler: Scalable storage for Hi-C data and other genomically labeled arrays. *Bioinformatics.* 2020;36(1):311–316. 10.1093/bioinformatics/btz540 31290943 PMC8205516

[ref-2] Allio R Schomaker-Bastos A Romiguier J : MitoFinder: Efficient automated large‐scale extraction of mitogenomic data in target enrichment phylogenomics. *Mol Ecol Resour.* 2020;20(4):892–905. 10.1111/1755-0998.13160 32243090 PMC7497042

[ref-3] Bernt M Donath A Jühling F : MITOS: Improved *de novo* metazoan mitochondrial genome annotation. *Mol Phylogenet Evol.* 2013;69(2):313–9. 10.1016/j.ympev.2012.08.023 22982435

[ref-4] Brůna T Hoff KJ Lomsadze A : BRAKER2: Automatic eukaryotic genome annotation with GeneMark-EP+ and AUGUSTUS supported by a protein database. *NAR Genom Bioinform.* 2021;3(1):lqaa108. 10.1093/nargab/lqaa108 33575650 PMC7787252

[ref-5] Challis R Richards E Rajan J : BlobToolKit - interactive quality assessment of genome assemblies. *G3 (Bethesda).* 2020;10(4):1361–1374. 10.1534/g3.119.400908 32071071 PMC7144090

[ref-6] Cheng H Concepcion GT Feng X : Haplotype-resolved *de novo* assembly using phased assembly graphs with hifiasm. *Nat Methods.* 2021;18(2):170–175. 10.1038/s41592-020-01056-5 33526886 PMC7961889

[ref-24] Di Tommaso P Chatzou M Floden EW : Nextflow enables reproducible computational workflows. *Nat Biotechnol.* 2017;35(4):316–319. 10.1038/nbt.3820 28398311

[ref-7] Goater B Senior G Dyke R : British Pyralid Moths.Colchester: Harley Books,1986. Reference Source

[ref-8] Guan D McCarthy SA Wood J : Identifying and removing haplotypic duplication in primary genome assemblies. *Bioinformatics.* 2020;36(9):2896–2898. 10.1093/bioinformatics/btaa025 31971576 PMC7203741

[ref-9] Harry E : PretextView (Paired REad TEXTure Viewer): A desktop application for viewing pretext contact maps. 2022; (Accessed: 19 October 2022). Reference Source

[ref-10] Heinrich C : Some new American Pyralidoid moths. *Proc Entomol Soc Wash.* 1939;42(2):33–34.

[ref-11] Howe K Chow W Collins J : Significantly improving the quality of genome assemblies through curation. *GigaScience.* Oxford University Press,2021;10(1):giaa153. 10.1093/gigascience/giaa153 33420778 PMC7794651

[ref-12] Kerpedjiev P Abdennur N Lekschas F : HiGlass: Web-based visual exploration and analysis of genome interaction maps. *Genome Biol.* 2018;19(1):125. 10.1186/s13059-018-1486-1 30143029 PMC6109259

[ref-13] Langmaid JR Young MR : Microlepidoptera Review of 2003. *The Entomologist’s Record and Journal of Variation.* 2004;116(5):193–214. Reference Source

[ref-14] Manni M Berkeley MR Seppey M : BUSCO Update: Novel and Streamlined Workflows along with Broader and Deeper Phylogenetic Coverage for Scoring of Eukaryotic, Prokaryotic, and Viral Genomes. *Mol Biol Evol.* 2021;38(10):4647–4654. 10.1093/molbev/msab199 34320186 PMC8476166

[ref-15] Neunzig HH : Moths of America North of Mexico, Fascicle 15.3 - Pyraloidea, Pyralidae Phycitinae (Part). *The Wedge Entomological Research Foundation.* 1990;165. Reference Source

[ref-16] Parsons MS Davis AM : Pyralidae.In: J.R. Langmaid, S.M. Palmer, and M.R. Young (eds) *A Field Guide to the Smaller Moths of Great Britain and Ireland.*The British Entomological and Natural History Society,2018.

[ref-17] Rao SSP Huntley MH Durand NC : A 3D map of the human genome at kilobase resolution reveals principles of chromatin looping. *Cell.* 2014;159(7):1665–80. 10.1016/j.cell.2014.11.021 25497547 PMC5635824

[ref-19] Rhie A McCarthy SA Fedrigo O : Towards complete and error-free genome assemblies of all vertebrate species. *Nature.* 2021;592(7856):737–746. 10.1038/s41586-021-03451-0 33911273 PMC8081667

[ref-18] Rhie A Walenz BP Koren S : Merqury: Reference-free quality, completeness, and phasing assessment for genome assemblies. *Genome Biol.* 2020;21(1):245. 10.1186/s13059-020-02134-9 32928274 PMC7488777

[ref-20] Simão FA Waterhouse RM Ioannidis P : BUSCO: assessing genome assembly and annotation completeness with single-copy orthologs. *Bioinformatics.* 2015;31(19):3210–2. 10.1093/bioinformatics/btv351 26059717

[ref-21] Streltzov AN Ustjuzhanin PY Yakovlev RV : Lepidoptera of South Ossetia (Northern Transcaucasia). Part I. Introduction and Superfamily Pyraloidea Latreille, 1809. *Acta Biologica Sibirica.* 2022;8:281–296. Reference Source

[ref-22] Surana P Muffato M Qi G : sanger-tol/readmapping: sanger-tol/readmapping v1.1.0 - Hebridean Black (1.1.0). *Zenodo.* 2023a; (Accessed: 17 April 2023). 10.5281/zenodo.7755665

[ref-23] Surana P Muffato M Sadasivan Baby C : sanger-tol/genomenote (v1.0.dev). *Zenodo.* 2023b; (Accessed: 17 April 2023). 10.5281/zenodo.6785935

[ref-25] Uliano-Silva M Ferreira JGRN Krasheninnikova K : MitoHiFi: a python pipeline for mitochondrial genome assembly from PacBio High Fidelity reads. *bioRxiv.* [Preprint],2022. 10.1101/2022.12.23.521667 PMC10354987 37464285

[ref-26] Vasimuddin M Misra S Li H : Efficient Architecture-Aware Acceleration of BWA-MEM for Multicore Systems.In: *2019 IEEE Int Parallel Distrib Process Symp (IPDPS).*IEEE,2019;314–324. 10.48550/arXiv.1907.12931

[ref-28] Wellcome Sanger Institute: The genome sequence of the Thicket Knot-horn, *Acrobasis suavella* (Zincken, 1818). European Nucleotide Archive.[dataset], accession number PRJEB52024,2022.

[ref-27] Zhou C McCarthy SA Durbin R : YaHS: yet another Hi-C scaffolding tool. *Bioinformatics.* Edited by C. Alkan,2023;39(1):btac808. 10.1093/bioinformatics/btac808 36525368 PMC9848053

