# Supplementary material for: The genome sequence of the Thicket Knot-horn, Acrobasis suavella (Zincken, 1818)
Source: Wellcome Open Res. 2023 Jun 19;8:252. [Version 1] doi: 10.12688/wellcomeopenres.19506.1 (PMC11303944; doi:10.12688/wellcomeopenres.19506.1)
Supplement: Peer review history

# Reviewer report: Reviewer response for version 1

**DOI:** 10.21956/wellcomeopenres.21608.r85321

**Published:** 2024-08-06

**Authors:** Sara Goodwin

**Affiliations:** 1 Cold Spring Harbor Laboratory, New York, USA

This report clearly describes the methods used and the quality of the genome assembly of the Thicket knot-horn. There is an extra % after 0.13% on page 3 column 2 paragraph 1. The authors should note which version of the PB library prep kits was used (express template prep kit 2.0? 3.0?). I have no additional comments.

Are sufficient details of methods and materials provided to allow replication by others?

Yes

Is the rationale for creating the dataset(s) clearly described?

Yes

Are the datasets clearly presented in a useable and accessible format?

Yes

Are the protocols appropriate and is the work technically sound?

Yes

Reviewer Expertise:

I am a next-generation sequencing expert with specific expertise in long-read sequencing.  In particular I work on cancer and plant genomes.

I confirm that I have read this submission and believe that I have an appropriate level of expertise to confirm that it is of an acceptable scientific standard.

# Reviewer report: Reviewer response for version 1

**DOI:** 10.21956/wellcomeopenres.21608.r85318

**Published:** 2024-06-17

**Authors:** Younghwan Kwak

**Affiliations:** 1 Department of Life and Environmental Sciences, University of California Merced, Merced, California, USA

**Is the rationale for creating the dataset(s) clearly described?**

 The background statement provides comprehensive details about the species *Acrobasis suavella, *including its physical characteristics, habitat preferences, larval food plants and geographical distribution. The statement also mentions the sequencing of the genome as part of the Darwin Tree of Life Project, which aims to sequence all named eukaryotic species in the Atlantic Archipelago of Britain and Ireland. 

 However, the rationale for the genome report could have been more explicitly described. While the inclusion in the Darwin Tree of Like Project provides a general context, the statement does not clearly explain why *A. suavella*, in parcticular, was chosen for genome sequencing. Mentioning specific reasons such as its ecological importance, potential for range expansion or its role in biodiversity studies would strengthen the rationale. 

**Are the protocols appropriate and is the work technically sound?**

 The protocols are appropriate and the work is technically sound. The detailed methodology and use of reputable tools and techniques support the validity and reliability of the study's results. 

 Specimens were appropriately collected and preserved, and DNA/RNA extractions were performed using standard, reliable methods. Sequencing on PacBio Sequel II and Illumina NovaSeq 6000 platforms ensured high-quality data. Genome assembly with Hifiasm, scaffolding with Hi-C data, and quality assessments with Merqury BolbToolKit and BUSCO confirmed the assembly's reliabilty. The BRAKER2 pipeline was appropriately used for genome annotation. 

**Are sufficient details of methods and materials provided to allow replication by others?**

 Overall, the comprehensive description of the methodologies, including the specific tools, software versions, and step-by-step procedures, ensures that other researchers can replicate the study's processes reliably. 

**Are the datasets clearly presented in a useable and accessible format?**

 The datasets are clearly presented in a usable and accessible format. I was able to access the data and confirm its availability.

Are sufficient details of methods and materials provided to allow replication by others?

Yes

Is the rationale for creating the dataset(s) clearly described?

Partly

Are the datasets clearly presented in a useable and accessible format?

Yes

Are the protocols appropriate and is the work technically sound?

Yes

Reviewer Expertise:

Entomology, Symbiosis, Insect-microbe interactions, genomics, transcriptomics

I confirm that I have read this submission and believe that I have an appropriate level of expertise to confirm that it is of an acceptable scientific standard.
